# A cardiac implantable device infection by *Raoultella planticola* in an immunocompromized patient

**DOI:** 10.1099/jmmcr.0.005080

**Published:** 2017-02-28

**Authors:** Chandra Adjodah, Chistophe D'Ivernois, David Leyssene, Jean-Baptiste Berneau, Yann Hemery

**Affiliations:** ^1^​ Infectious and Tropical Diseases Department, Amiens University Hospital, Amiens, France; ^2^​ Department of Cardiovascular Diseases, Basque Coast Hospital Centre, Bayonne, France; ^3^​ Department of Microbiology, Basque Coast Hospital Centre, Bayonne, France

**Keywords:** implantable cardiac device infection, *Raoultella planticola*, immunosuppression, methotrexate, corticosteroids, rheumatoid arthritis

## Abstract

**Introduction.** Infection of cardiac implantable electronic devices is a severe condition associated with high mortality, particularly in patients who are dependent upon heart-pacing devices. *Staphylococci* are found in 70 % of reported cases.

**Case presentation.** We report the case of a cardiac-pacemaker infection in a 79-year-old man, cumulating a history of rheumatoid arthritis treated by corticosteroids and methotrexate by a recently identified micro-organism: *Raoultella planticola*. He presented local signs of infection on his VVI pacemaker implantation site and underwent urgent pocket device replacement under cefamandole antibioprophylaxis. On incision thick pus oozed out. It was necessary to perform a complete hardware extraction comprising the pulse generator and the ancient lead. Pus was inoculated into aerobic and anaerobic culture vials and Gram staining unveiled Gram-negative rods. Microbiology analysis identified the organism as *R. planticola.* A new pacing device was inserted on the contrlateral pectoral region. Ciprofloxacin enabled full recovery. A literature review concerning this pathogen revealed that it is involved in severe infections such as bloodstream infections, peritonitis, cellulitis, pneumonia and lung abscesses, and urinary tract infections. In these case reports, underlying co-morbidities were identified such as solid active neoplasia, recent chemotherapy, corticosteroids, solid-organ-recipient patients and recent open surgery.

**Conclusion.** *R. planticola* is a serious emerging pathogen and contributes to the burden of various infectious conditions. Its pathogenicity and occurrence should be known by clinicians and a high level of awareness is necessary to precisely identify it provide the correct antibiotic regimen.

## Introduction

Cardiac-device infections constitute a real burden associated with a high mortality rate particularly in the elderly. Bacteria other than staphylococci are rarely involved. *Raoultella planticola*, a Gram-negative bacillus, is known as an infective agent in a few reported cases, particularly in immunocompromized patients. It is a newly identified bacterium, closely related to *Klebsiella* spp. In world where antibiotic resistance is a great menace, the pathogenicity and phylogenic characteristics of *R. planticola* should be known by clinicians so that the adequate and optimal antibiotic regimen be prescribed.

## Case report

We report the case of a 79-year-old male patient who presented a swollen and painful pacemaker implantation site on the left pectoral region since 6 days. Symptoms included erythema, warmth, fluctuance and tenderness around the pocket generator without wound dehiscence and purulent drainage. His body temperature was 37.8 °C with neither reported fever nor chills. No cardiac murmur was heard and a thorough skin examination revealed neither Osler nodes nor erythematous lesions which could suggest sub-acute bacterial endocarditis. Lung and pleural auscultation was non-contributive. Abdominal palpation revealed the absence of spleen enlargement and no enlarged lymph nodes. His history was consequent: rheumatoid arthritis treated by current corticosteroids (0.8 mg kg^−1^ daily) and methotrexate (12.5 mg daily), peripheral arterial disease with multiple arterial bypass grafts on the lower limbs in 1980, a stroke in 2007, and prostate cancer with recent partial prostatectomy and ongoing hormonotherapy.

He had been implanted in 2004 on the left pectoral region with a VVI pacemaker for permanent atrial fibrillation with slow ventricular rate. The cardiac stimulator applied a non-bi-ventricular pacing regimen with a barbed electrode lead implanted in the right ventricle. The pulse generator has been changed in 2009 under cefazolin antibioprophylaxis for battery depletion and the postoperative course has been uneventful. On admission, the patient was afebrile with a blood count revealing 10 400 leucocytes with a predominance of neutrophils (78 %), a C reactive protein level of 88 mg l^−1^, a normalized international ratio of 2.46 and a serum creatinine level at 142 µM. His electrocardiogram revealed permanent ventricular pacing without spontaneous escape rhythm. Transoesophageal ultrasound confirmed the absence of vegetation on the heart valves and on the electrode leads.

The patient underwent urgent pocket device replacement under cefamandole antibioprophylaxis with the aim of implanting a new VVI pacemaker on the right side and a lead screwed on the high ventricular septum as he was dependent upon the stimulator. The initial strategy was to remove the infected pocket by cutting the old electrode lead under the collarbone in a non-septic zone and leaving the extremity of the old lead in the non-infected area. Nevertheless when incising on the left subclavian side, thick pus with a fetid odour oozed out and it was necessary to perform a complete hardware extraction comprising the pulse generator and the old lead. Great care was taken when removing the whole material, generator plus electrode lead to prevent cardiac tamponade. Pus was actively drained and pyogenic samples were collected on dry swabs for Gram staining and culture in the laboratory. Pus was also inoculated into aerobic and anaerobic blood culture vials. Infected tissue was excised and debrided and the wound washed with normal saline and povidone-iodine. Afterwards, the wound was closed with separated Blair–Donati sutures. A new pacing device was inserted on the contrlateral pectoral region during the same procedure in this dependent patient as temporary epicardial pacing would have needed an urgent transfer to a reference centre which was not possible during this decisive intervention.

An antibiotic regimen comprising intravenous oxacillin, 9 g daily, targeting staphylococcal species and oral ciprofloxacin, 500 mg twice daily was administered. The patient presented a single feverish bout at 38.8 °C six hours after the replacement of the generator pocket and a series of blood cultures were carried out that turned out to be sterile after 4 days of culture. He remained afebrile afterwards.

## Investigations

Gram staining on pus unveiled the presence of Gram-negative bacilli. After 43 h of incubation, both the aerobic and anaerobic vials were productive after pus inoculation. The pus cultures in the aerobic and anaerobic media revealed the overgrowth of a single bacterium species on MacConkey no. 3 agar, which was identified as *R. planticola* using the VITEK technique based on biochemical profiles in liquid medium with a probability of 95 % (VITEK 2; bioMérieux). With respect to the antimicrobial susceptibility, the strain presented an intrinsic natural resistance pattern with a low-level penicillinase: resistant to ampicillin and ticarcillin, sensitive to amoxicillin/clavulanic acid (MIC ≤2 mg l^−1^) and piperacillin/tazobactam (MIC ≤4 mg l^−1^), sensitive to cefalotin (MIC ≤2 mg l^−1^), cefoxitin (MIC ≤4 mg l^−1^), cefotaxime (MIC ≤1 mg l^−1^) and ceftazidime (MIC ≤1 mg l^−1^), sensitive to all penems (MIC ≤0.5 mg l^−1^), sensitive to all aminoglycosides (kanamycin, tobramycin, gentamicin and amikacin: MIC ≤1–2 mg l^−1^), and sensitive to nalidixic acid and other quinolones (ciprofloxacin, ofloxacin, levofloxacin: MIC ≤ 0.25 mg l^−1^).

## Treatment

The antibiotic regimen was decremented as soon as the antibiogram was received to oral ciprofloxacin 500 mg twice daily for a duration of 15 days.

## Outcome and follow-up

The outcome was simple. The patient remained afebrile with no sign of systemic symptoms. The VVI cardiac device implanted on the right pectoral region remained fully functional with a permanent ventricular pacing without spontaneous escape rhythm. A transoesophageal ultrasound was carried out 2 weeks after the cardiac-device replacement which confirmed the absence of vegetation on the new lead. The patient was seen in the out-patient department 5 years later by the cardiologist. His health had remained uneventful.

## Discussion

We report the case of a cardiac-device infection by an unusual bacterium that had not been previously reported. The infection was initially suspected to be limited to the pocket of the device but the device had to be fully extracted including the lead. Microbiology analysis identified the pathogen as *R planticola* and susceptibility testing allowed an efficient monoantibiotic regimen by a fluoroquinolone to be used.

Cardiac-device infections are due to staphylococci in 70 % of cases in most reported series [1, 2] among which coagulase-negative being staphylococci predominant [3]. Meticillin resistance among these staphylococci is more frequently encountered among patients who have been in contact with healthcare environments [4, 5]. With respect to the occurrence of Gram-negative bacteria, *Candida* spp., *Corynebacterium* spp. and *Propionibacterium acnes,* the latter are not as frequent in cardiac-device infections [2, 3, 6].

*R. planticola* was identified as the pathogen in this cardiac-device infection. A literature review concerning this pathogen revealed that it is involved in severe infections such as bloodstream infections, peritonitis, cellulitis, pneumonia, lung abscesses and urinary tract infections. Nonetheless, we did not find any reports of implantable-device infections. Puerta-Fernandez *et al.* [7] described a *R. planticola* bacteraemia secondary to gastroenteritis in a patient who presented fever, severe abdominal pain and diarrhoea after ingesting poorly prepared fish. Other cases of infection due to this bacteria have been reported, including pneumonia and bacteraemia after a native valve surgery as described by Freney *et al.* [8], necrotizing pancreatitis with retroperitoneal abscess cultures as described by Alves *et al.* [9], a case of cholangitis with septic shock in an immunocompromized patient who underwent retrograde cholangiography as described by Yokota *et al*. [10], a case of soft tissue infection with cellulitis as described by O′Connell *et al.* [11], and an acute prostatitis in a case of a solid-organ-transplant recipient as described by Koukoulaki *et al.* [12]. In these case reports, underlying co-morbidities were identified such as solid active neoplasia, recent chemotherapy, corticosteroids, solid-organ-recipient patients, recent open surgery and retrograde cholangiopancreatography.

*Raoultella* spp. was first classified in the *Klebsiella* species in 2001 after comparative phylogenetic studies of the sequences of the 16S rRNA and *rpoB* genes by Drancourt *et al*. [13]. The genus *Raoultella* includes *R. planticola*, *R. ornitholytica*,‘*R. trevisanii*’ and *R. terrigena* [14], grows at 10 °C and uses l-sorbose as its carbon source. It is an environmental and telluric bacterium and is rarely found in human infections. It is an encapsulated, aerobic and non-motile Gram-negative bacillus. It is difficult to cultivate and to isolate by routine methods and is often mistaken for *Klebsiella* spp. It can ferment glucose with gaseous production. It has negative oxidative, positive catalase and negative alcohol dehydrogenase properties. Most strains can metabolise citrate and glucose, produce indole and assimilate histamine.

Our patient cumulated several risk factors including renal failure, corticosteroid use and methotrexate therapy for auto-immune disease, prostate cancer and anticoagulation use [15–17]. These conditions may have contributed to the cardiac-device infection. We have not been able to determine the entry point of the pathogen. Was it from the gut? Digestive endoscopy, however, revealed no tumour of the upper and lower digestive system. Either, there was no history of raw fish or prawn consumption. Furthermore, the use of corticosteroids and methotrexate could have contributed to mask fever rendering the detection of clinical signs of infection more difficult.

The minimal inhibitory concentrations values used come from the Antibiotic Committee of the French Society of Microbiology [18]. Matrix-associated laser desorption ionization-time of flight MS was not yet available to confirm the strain of *R. planticola*.

To the best of our knowledge, this is the first case of a cardiac-device infection by *R. planticola*. The underlying immunocompromized condition of the patient could have contributed to the infection although the entry point of the pathogen is unclear.

*R. planticola* is a serious emerging pathogen and contributes to the burden of various infectious conditions. Its pathogenicity and occurrence should be known by clinicians and a high level of awareness is necessary to precisely identify it so as to provide the correct antibiotic regimen.
